# 84 Methotrexate toxicity in a patient with Takayasu arteritis; a case report

**DOI:** 10.1093/rheumatology/keac496.080

**Published:** 2022-10-07

**Authors:** Athul Kooliyath, Angela Migowa

**Affiliations:** The Aga Khan University, Nairobi

## Abstract

**Background:**

Methotrexate is an antimetabolite commonly used as a chemotherapeutic and as a steroid sparing agent in multiple rheumatologic conditions. The common side effects are well documented and usually seen in patients receiving high dose therapy and hence more commonly seen in oncology. Here, we discuss a case on methotrexate toxicity in a patient being treated for Takayasu arteritis.

**Methods:**

This was a retrospective case review

**Results:**

A seventeen-year-old was seen in the rheumatology service with a diagnosis of Takayasu arteritis after being initially worked up due to hypertension. Methotrexate therapy was started at an initial dose of 25 mg to be taken weekly. She was later seen in the pediatric out-patient unit 10 days post initiation of therapy with a history of inability to feed due to oral sores and severe abdominal pain. She also complained of odynophagia and hence had poor oral intake. On further history, it was noted that the patient was taking the medication on a daily basis as opposed to the prescribed weekly regimen. On exam, she was noted to have extensive ulceration of her buccal mucosa, gingiva and tongue. She was also found to have severe epigastric tenderness. Her genital regions were however spared. A diagnosis of methotrexate toxicity was made and she was admitted to the high dependency unit for management. Methotrexate was immediately discontinued and a hyper hydration regimen was initiated with intravenous fluids (125 ml/m^2^/h). Her pain was managed with an intravenous morphine infusion. Particular care was taken to avoid drugs such as proton pump inhibitors (PPIs), non-steroidal anti-inflammatory drugs (NSAIDs) and Sulphur containing drugs. A rescue was initiated with 15 mg/m^2^ of folinic acid given 6 hourly. The patient made a full recovery and was discharged 4 days later.

**Discussion and Conclusions:**

Methotrexate is a drug with varied and severe adverse effects. Toxicity with methotrexate affects multiple systems and can cause end organ damage. It is therefore of paramount importance to recognize toxicity early and manage appropriately before onset of end organ damage. The above patient was risk of hepatotoxicity, renal toxicity, Neurologic toxicity, methotrexate induced lung injury, hematologic toxicity and hypersensitivity. She developed mucositis as a result of the medication.

Enhancing renal elimination of the drug is at the core of management. This can be done with hyperhydration, alkalinisation of urine and avoiding drugs that slow elimination of methotrexate such as PPIs, NSAIDs, tyrosine kinase inhibitors, sulfa drugs and penicillin like drugs. Once end organ damage is established, therapy is supportive and highly dependent on the organ systems involved and the extent of injury.

It is necessary for clinicians to be well versed with the adverse effects expected with methotrexate as evidenced by this case for prompt diagnosis and sound management. It is also vital to counsel patients and parents about the medication- correct dosage and timing, expected adverse effects and when to seek medical attention.

